# Supplementary material for: CLSY docking to Pol IV requires a conserved domain critical for small RNA biogenesis and transposon silencing
Source: Nat Commun. 2024 Nov 27;15:10298. doi: 10.1038/s41467-024-54268-0 (PMC11603163; doi:10.1038/s41467-024-54268-0)
Supplement: Supplementary file 1 — Supplementary Information [file 41467_2024_54268_MOESM1_ESM.pdf]

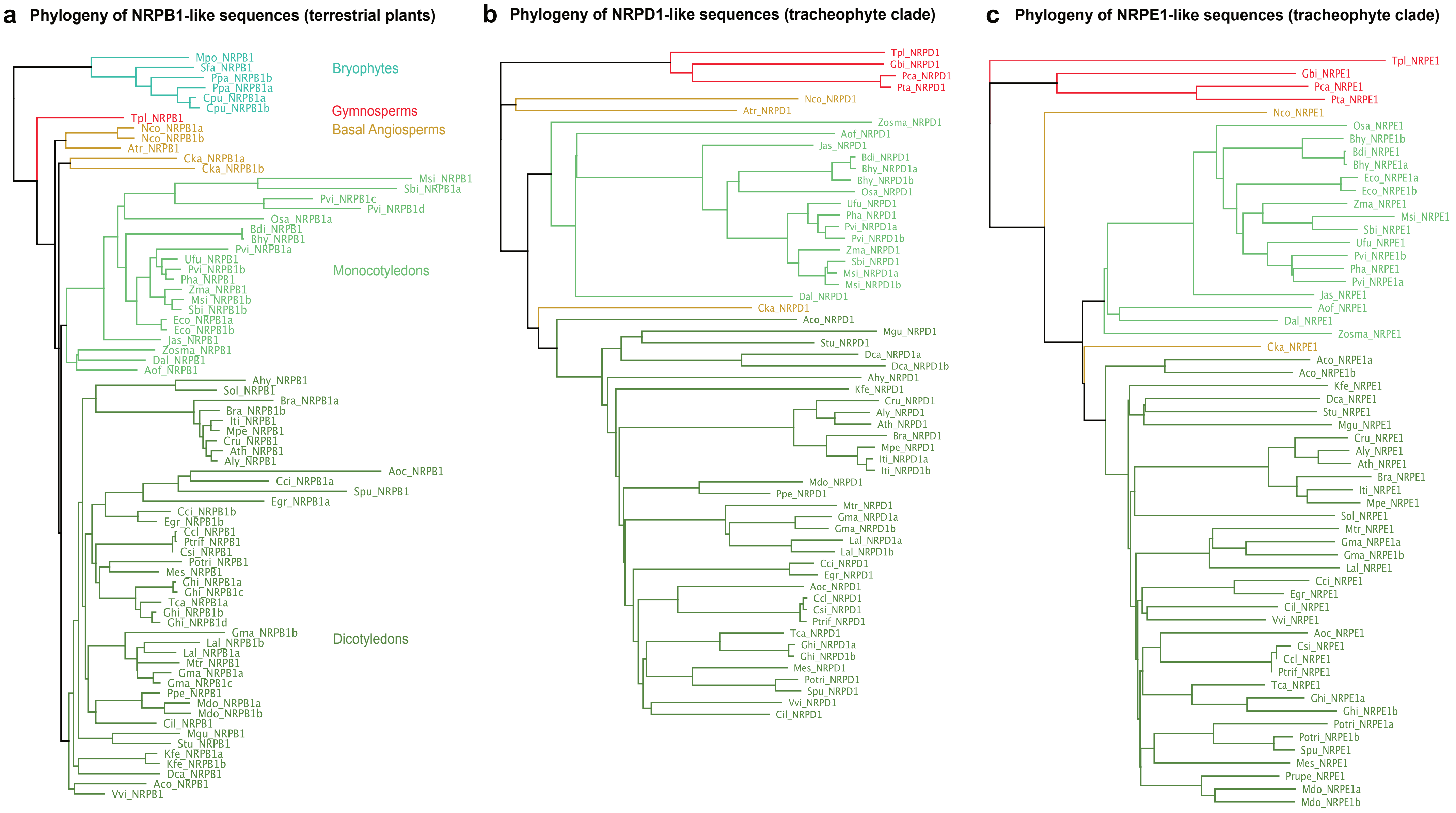

Supplementary Figure 1. Phylogenetic analyses of the largest subunits of Pol II, Pol IV and Pol V in terrestrial plants. Phylogenetic tree representing available **(a)** NRPB1, **(b)** NRPD1 and **(c)** NRPE1 proteins from diverse terrestrial plants. The tree covers terrestrial plant species including gymnosperms and diverse angiosperms (basal, monocotyledons, and dicotyledons). The protein sequences were obtained from the Phytozome13 and NCBI databases. The phylogenetic analyses were performed with the Geneious software package using MUSCLE for multiple sequence alignment and the Geneious neighbor-joining tree builder with the Jukes-Cantor distance model. The full list of RNA polymerase subunits used in this analysis (including the corresponding species names, database accessions and protein characteristics) is provided in **Supplementary Data 1**.

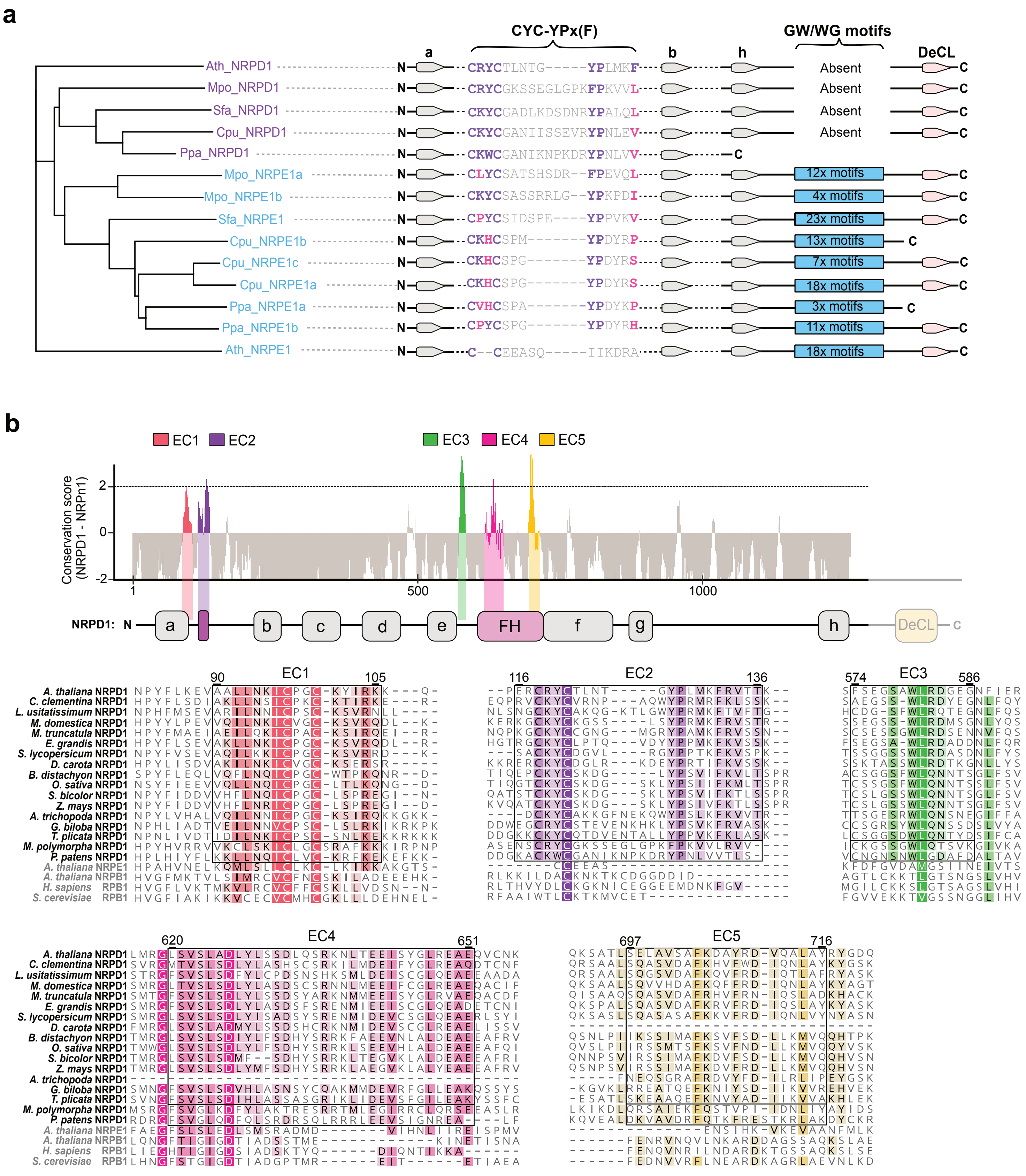

Supplementary Figure 2. Bryophyte NRPD1 and NRPE1 proteins have divergent CYC-YPxF motifs as compared to NRPD1 in vascular plants. (a) Phylogenetic tree of the CYC-YPxF region in bryophyte NRPD1 and NRPE1-like proteins compared to corresponding *A. thaliana* orthologs. Putative bryophyte NRPD1 subunits (Pol IV) are indicated in purple and putative NRPE1 subunits (Pol V) are indicated in light blue. Bryophyte NRPE1-like protein identities were inferred in this analysis based on GW/WG repeat sequences found in the C-terminal domain (CTD) of each protein. (**b**) ***top panel:*** Reproduction of the **Fig. 1d** ConSurf analysis of NRPD1 proteins from 52 vascular plant species compared to NRPB1 and NRPE1. The five protein regions that are peaks of NRPD1-exclusive conservation (EC1-EC5) are plotted in color along the subunit's domain architecture. ***bottom panels:*** Exclusively conserved (EC) regions are marked by boxes in the multiple sequence alignments of NRPD1 proteins of diverse species, with conserved residues in colors matching the distinct EC regions. The amino acid numbering above the boxed EC regions is based on NRPD1 of *A. thaliana*. Corresponding NRPD1 regions from two representative bryophytes, *Marchantia polymorpha* and *Physcomitrium patens*, are boxed separately to show how the Pol IV-specific motifs have diverged in non-vascular plants. Positions and domains with low NRPD1 exclusive conservation are in grey.

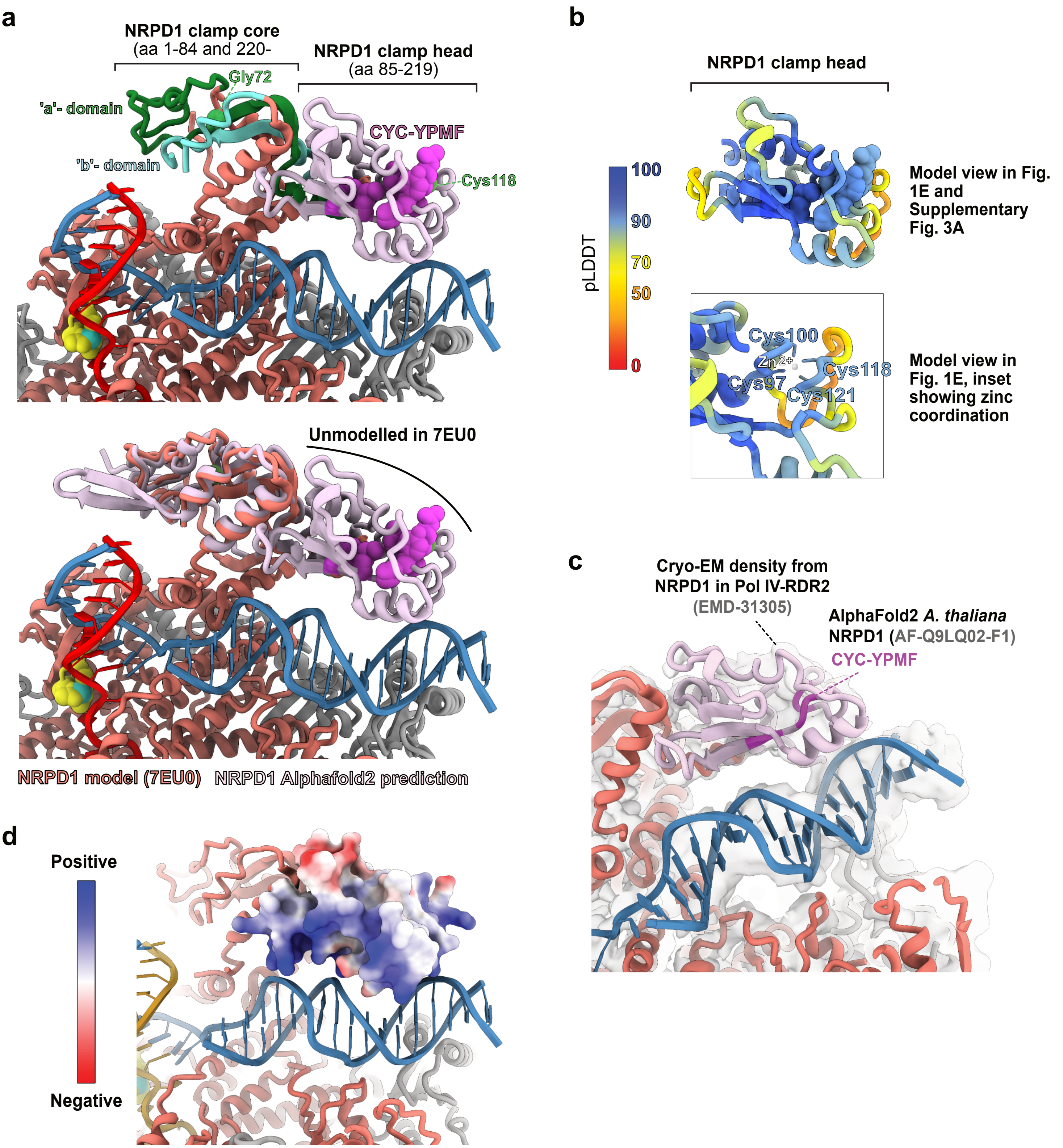

Supplementary Figure 3. Structural analysis and validation of the Pol IV clamp head model. (a) AlphaFold2 prediction of the previously unmodelled 'clamp head' domain structure of NRPD1 that contains the CYC-YPMF residues (bright purple) and is located close to the DNA template (blue). ***top panel:*** an AlphaFold2 NRPD1 N-terminus prediction (aa 1-298) was fit into the NRPD1 'clamp core' structure to form an extended Pol IV model: aa 1-84 and 220-319 (clamp core) derive from the 7EU0 structure of NRPD1, while aa 85-219 are from the AlphaFold2 prediction of NRPD1 and correspond to the clamp head (light pink). The NRPD1 positions Gly72 and Cys118 (green) are mutated in the *nrpd1-49* and *nrpd1-50* mutants, respectively[41]. ***bottom panel:*** the 7EU0 structure of NRPD1 (coral) is superimposed with the AlphaFold2 prediction of the full NRPD1 clamp (pink). **(b)** ***top panel:*** The CYC-YPMF-containing clamp head domain is shown as predicted by AlphaFold2 with colors representing AlphaFold2's per-residue confidence score (pLDDT). ***bottom panel:*** pLDDT for the AlphaFold2 clamp head prediction including the Cys97-Cys100-Cys118-Cys121 residues coordinating zinc and with aa 166-172 removed to avoid obscuring the view of the cysteine side chains. Residues with pLDDT >90 are expected to be modelled to high accuracy, scores of 70-90 are expected to be well-modelled (good backbone), scores of 50-70 are low confidence, and scores of 0-50 should not be interpreted (likely disordered)[74]. **(c)** To check for the presence of the NRPD1 clamp head domain, the cryo-EM density of the Arabidopsis Pol IV-RDR2 complex (EMD-31305) was inspected. The map was low-pass filtered to expose regions of lower resolution, which confirmed that the CYC-YPMF-containing domain is present. **(d)** The AlphaFold2 predicted NRPD1 clamp head domain is displayed according to its Coulombic electrostatic potential (ESP), with the color ranging from red for negative potential to blue for positive potential. The DNA facing part of the domain is largely positively charged, which is expected as DNA is largely negatively charged. For this analysis, the low confidence loop formed by residues Lys105 to Gln114 was trimmed because it was clashing with the DNA template.

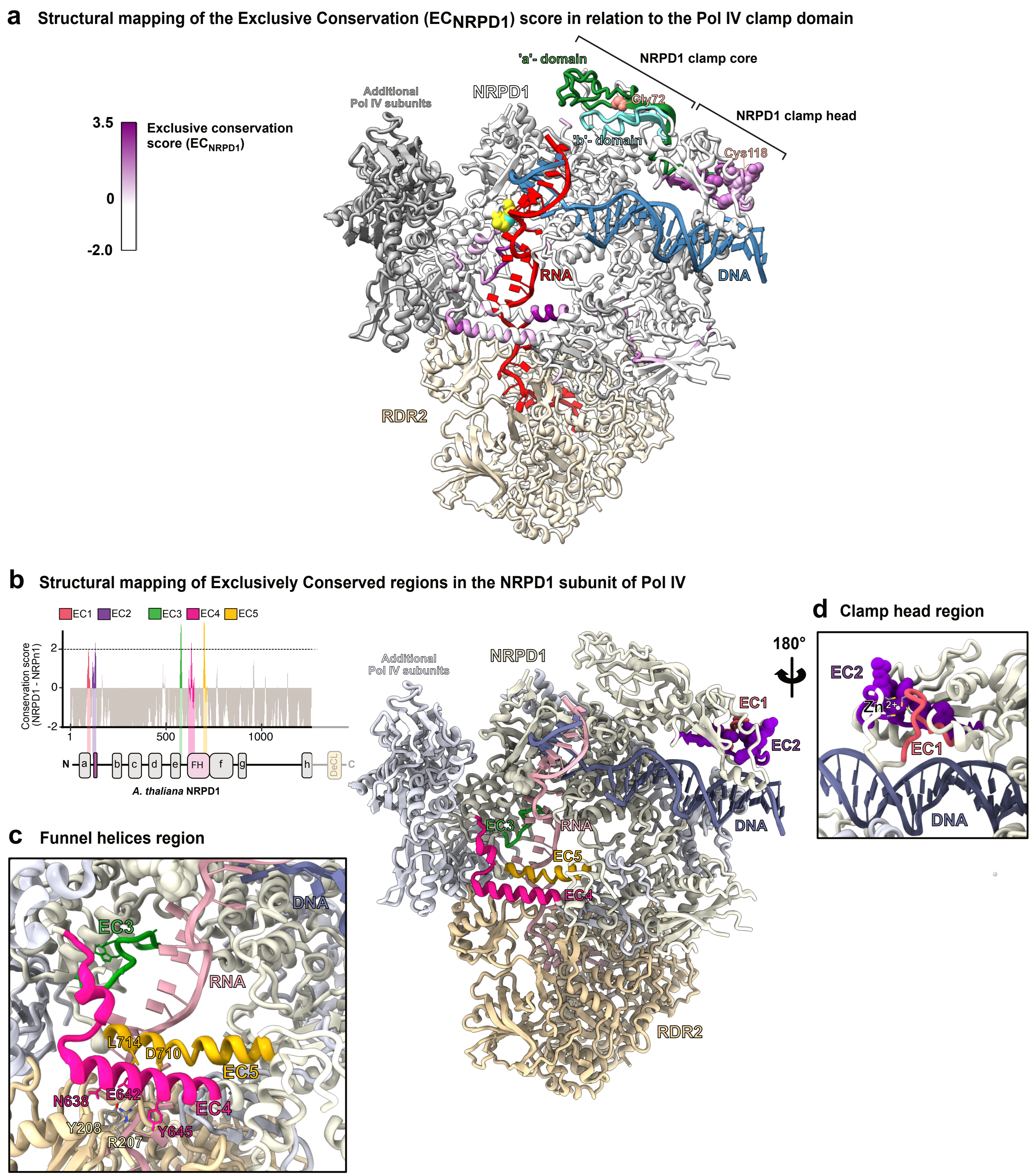

Supplementary Figure 4. Structural representation of the exclusively conserved regions in the largest subunit of Pol IV (NRPD1). (a) Structural mapping of the NRPD1 exclusive conservation score (EC_{NRPD1}, sliding average) rendered as a purple color gradient on the white NRPD1 amino acid chain. The DNA template is shown in blue, the transcript product of Pol IV in crimson, and the active site in yellow. RDR2 is shown in light beige, whereas NRPD2 and most other Pol IV subunits are not shown. The universally conserved RNA polymerase ‘a’ and ‘b’-domains of NRPD1 are shown in green and turquoise, with the clamp core and clamp head domains indicated with brackets **(b)** The Pol IV-RDR2 model of **Fig. 1e** but with the five peaks of NRPD1-exclusive conservation (EC1-EC5) plotted in color along the amino acid chain in the 3D model. **Fig. 1d** is reproduced at left as a key for comparing the universally conserved (‘a’-‘h’) domains to the funnel helices and EC regions. **(c)** Zoom inset of the NRPD1 funnel helices (EC4 and EC5) that interact with RDR2, as well as the EC3 loops that are adjacent to the Pol IV RNA transcript as it enters RDR2. Individual NRPD1 amino acids known to contact RDR2 (N638, E642, Y645, D710 and L714)[27] are shown in bright pink and gold, indicating the EC4 and EC5 regions, respectively. Two amino acids from RDR2 that make contact with NRPD1 (R207 and Y208)[27] are labelled in light beige. The NRPD1 amino acid positions covered by EC1-EC5 and relevant functional domains are provided in **Supplementary Table 3. (d)** Zoom inset of the NRPD1 clamp head modeled using AlphaFold2, rotated 180 degrees with respect to the main model view and showing EC1 and EC2 regions, as well as Cys97-Cys100-Cys118-Cys121 coordinating zinc.

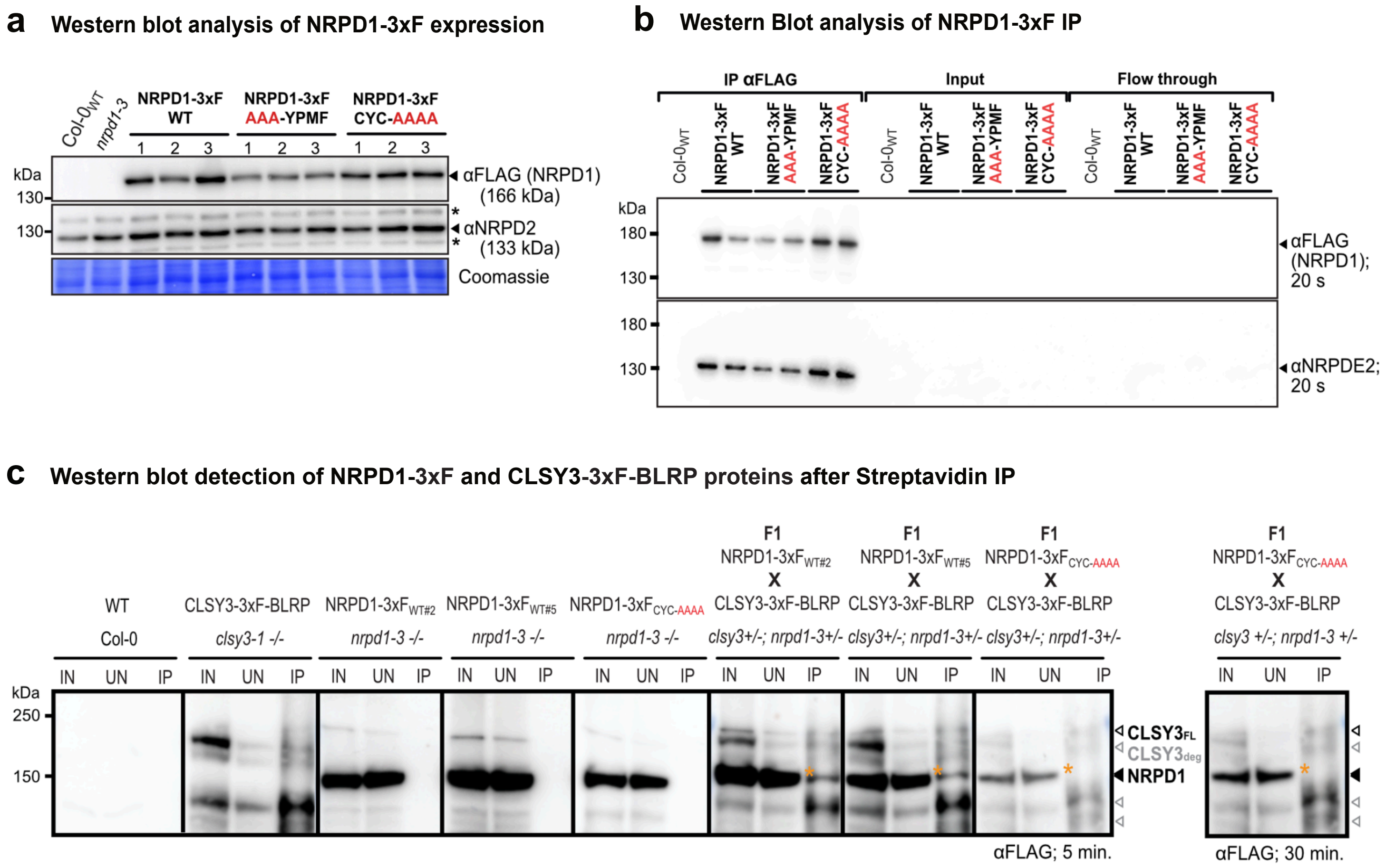

Supplementary Figure 5. Western blot validation of Arabidopsis lines used for Pol IV complex IP-MS analyses. (a) Western blots of the NRPD1-3xF and NRPD2 subunits of Pol IV in extracts from Col-0_{WT} and *nrpd1-3* mutant flowers (negative controls) compared to independent insertion lines (1,2,3) expressing NRPD1-$3xF_{WT}$, NRPD1-$3xF_{AAA\text{-}YPMF}$ or NRPD1-$3xF_{CYC\text{-}AAAA}$ variants in the *nrpd1-3* background (see **Fig. 2a**). **(b)** Western blots of the NRPD1-3xF variants and NRPD2 in the anti-FLAG immunopurification experiments analyzed by liquid chromatography-tandem mass spectrometry (see **Fig. 2b, c, d**). The anti-FLAG immunopurified, input and column flow-through aliquots were loaded onto a single gel for comparison. Because of the limited wells available in the SDS-PAGE gel, this western analysis only includes the first two biological replicates of each NRPD1-3xF variant represented in **Fig. 2b**. **(c)** Anti-FLAG western blot detecting NRPD1 and CLSY3 proteins from either input (IN), unbound (UN), or streptavidin IP (IP) samples from the genotypes indicated above each blot. Protein sizes are indicated in kDa on the left and the bands corresponding to NPRD1 as well as both full length CLSY3 ($CLSY3_{FL}$; Black) and several CLSY3 degradation products ($CLSY3_{deg}$; Grey) are indicated on the right. The orange asterisk marks the location of the band representing the co-IP of NRPD1 with CLSY3. A longer exposure (30 min.) is shown to assess NRPD1 enrichment in the IP lane when similar levels of NRPD1 protein are present in the IN and UN lanes as observed for the NRPD1-$3xF_{WT\#2}$ and NRPD1-$3xF_{WT\#5}$ lines. Source data are provided as a Source Data file.

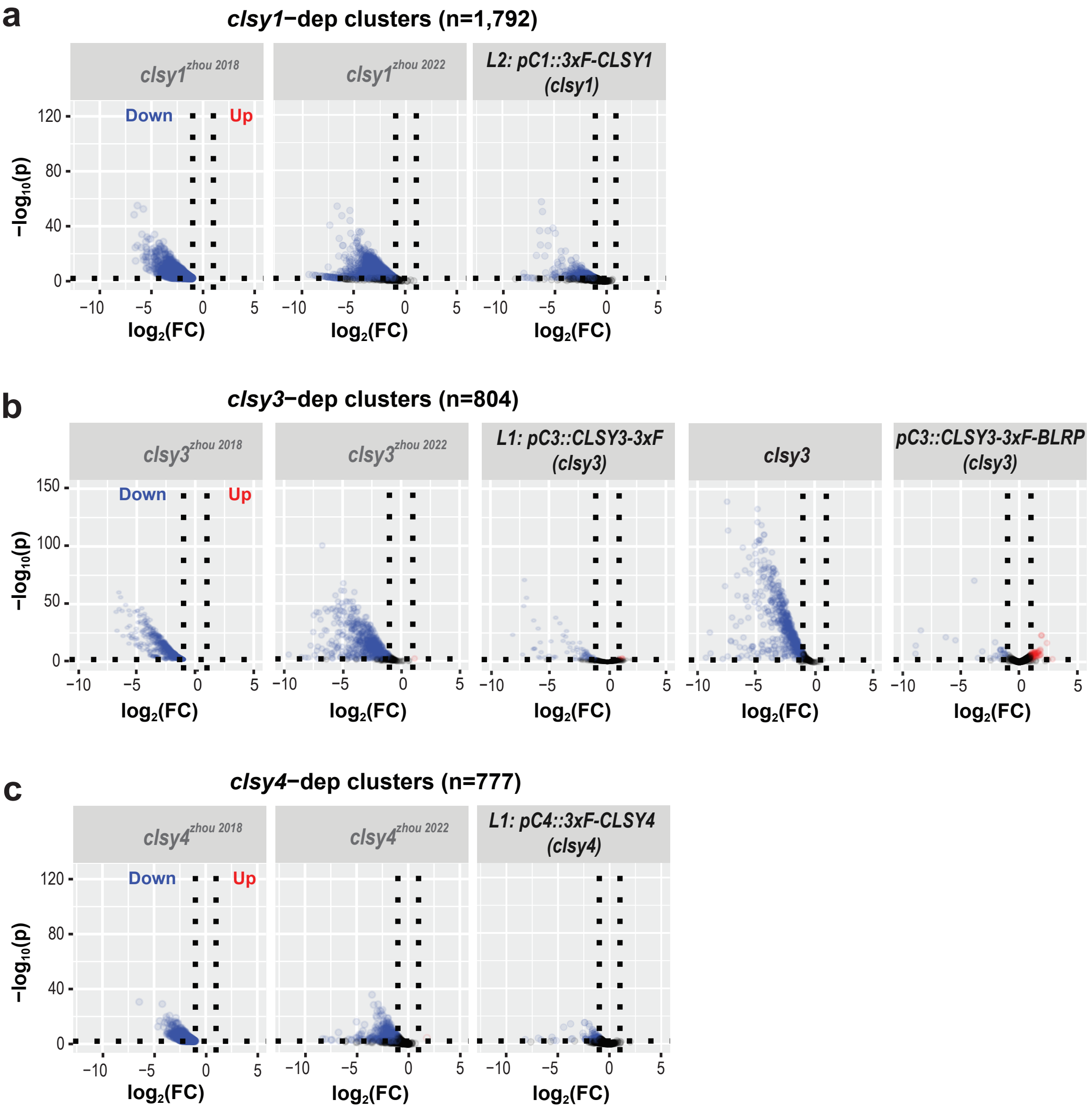

Supplementary Figure 6. Complementation of *clsy* mutants with 3xF-tagged CLSY transgenes. (**a**-**c**) Volcano plots showing siRNA levels at *clsy1*-, *clsy3*-, and *clsy4*-dependent clusters (n=1792, 804, and 777, respectively), as defined in Zhou et al. 2018[11]. For each plot, clusters that are downregulated compared to wild-type controls (log_2 FC ≤ -1 and FDR ≤ 0.01) are shown as blue circles, those unaffected are shown as black circles, and those upregulated (log_2 FC ≥ 1 and FDR ≤ 0.01) are shown as red circles. For all panels, the plots with genotypes indicated in dark grey show previously published control data from Zhou et al. 2018[11] and 2022[21]. The remaining plots show siRNA levels in the indicated *clsy* mutants assayed in parallel with previously unpublished lines from Zhou et al. 2022[21] (L2: pC1::3xF-CLSY1, L1: pC3::CLSY3-3xF, and L1: pC4::3xF-CLSY4) and from a new unpublished sRNAseq dataset (pC3::CLSY3-3xF-BLRP). In each case, the *CLSY* genes are driven by their endogenous promoters (pC#) and the line number (L#) and N or C-terminal position of the 3xF tag is as indicated.

Supplementary Figure 7 (next page). Assessment of siRNA levels in various *nrpd1* mutant and NRPD1-3xF variant lines. (**a**) Pie charts showing the fraction of 24 nt siRNAs from the indicated control samples that fall inside (included) or outside (excluded) of the 12,939 clusters defined in Zhou et al. 2022[21]. (**b**) Volcano plots showing 24 nt siRNA levels at the full set of clusters (n=12,939) defined in Zhou et al. 2022[21]. For each plot the genotype is indicated at the top and clusters that are downregulated compared to wild-type controls (log_2FC ≤ -1 and FDR < 0.01) are shown as blue circles, those unaffected are shown as black circles, and those upregulated (log_2 FC ≥ 1 and FDR < 0.01) are shown as red circles. The numbers of clusters in each category are indicated in the correspondingly colored boxes and a boxplot showing the overall effect of each mutant is included along the x-axis. (**c**) Northern blot analysis comparing small RNA levels in the indicated genotypes using probes for several RdDM targets (*LTR META1, AtREP2*, *AT1TE53395, SIMPLEHAT2* and *siRNA02*) or for miR160*,* as a loading control. (**d**) Scaled Venn diagrams depicting reduced siRNA clusters identified using flower tissue in the indicated mutants as originally published in Zhou et al. 2022[21], except the numbers correspond to the total number of reduced siRNA clusters in each mutant rather than those unique to each mutant. (**e**) Violin plots showing the normalized expression levels [log_2 (rpkm +1)] of 24nt siRNA clusters in the indicated categories and genotypes. Replicate samples are designated as r1 and r2. Select genotypes are shown on a smaller Y-axis scale on the right. The less opaque upper panel is reproduced from **Fig. 4a**. Source data are provided as a Source Data file.

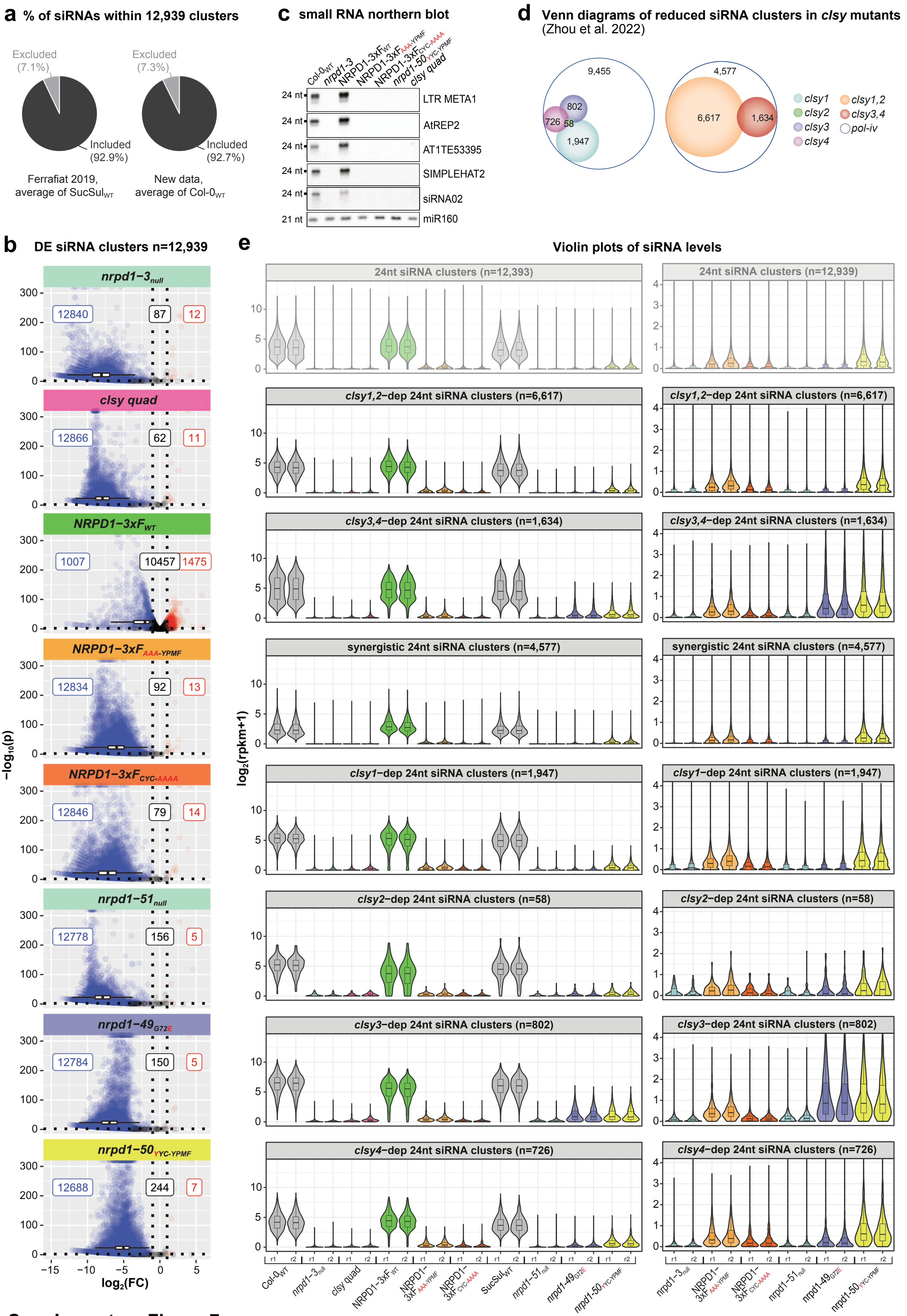

Supplementary Figure 7.

Supplementary Table 1. Oligonucleotide primer and probe sequences.

Genotyping

Experiment	Oligo_name	Sequence
genotyping for SALK_083051	nrpd1-4-geno-RP	AACCTCGAAGCAACAAGAATCTCCG
	nrpd1-4-geno-LP	ATCACGGTGACTGTAGTTGAAGCC
	Salk primer LBa1	TGGTTCACGTAGTGGGCCATCG
genotyping for SALK_018319	clsy1-7-geno-RP	TTTCTCGCGAGCTACTTGAAG
	clsy1-7-geno-LP	AAAAGCTCCTGAGGGTTGAAG
	Salk primer LBa1	TGGTTCACGTAGTGGGCCATCG
genotyping for SALK_040366	clsy3-1-geno-LP	CTTCTTGCAGTGGCATTCTTC
	clsy3-1-geno-RP	ATTAGGCGAAGAGGATGAAGC
	Salk primer LBa1	TGGTTCACGTAGTGGGCCATCG
genotyping for SALK_003876	clsy4-1-geno-LP	TTGACCGTCGTTTTTCTTCAG
	clsy4-1-geno-RP	GCTCCATGATCAGCTTCAGAG
	Salk primer LBa1	TGGTTCACGTAGTGGGCCATCG
check the presence of the Flag N-terminal for 3xFlag_CLSY1	BLRP_F	TGGCTGGTGGACTTAACGAT
	CLSY1_reverse	GAGACGCATTGTCATCGTTC
check the presence of the Flag C-terminal for CLSY3_3xFlag	CLSY3 forward	TGCATGTTCTTCCAGACATG
	BLRP_R	CGAAGATATCGTTAAGTCCACCA
check the presence of the Flag C-terminal for CLSY4_3xFlag	JL2359	CGAACTGGTCTTCTCTTCTAC
	BLRP_R	CGAAGATATCGTTAAGTCCACCA
check the presence of the Flag N-terminal for 3xFlag_CLSY4	BLRP_F	TGGCTGGTGGACTTAACGAT
	JL2357	ACAAGCATCACGCATGTTAAC

Probes for small RNA blot

Target	Oligo_name	Sequence
LTR_META1 siRNA	LTR_META1_probe	GCCCATCATCTAAGCCCATCATCT
AtREP2 siRNA	AtREP2_probe	GCGGGACGGGTTTGGCAGGACGTTACTTAAT
AT1TE53395 siRNA	AT1TE53395_probe_F	ATTCTCTTCTACTTTGACCTACTTTCG
	AT1TE53395_probe_R	TGCTCTTCAATGGTGGGCTC
SIMPLEHAT2 siRNA	SIMPLEHAT2_probe	TGGGTTACCCATTTTGACACCCCTA
siR02 siRNA	siR02_probe	GTTGACCAGTCCGCCAGCCGAT
miR160 siRNA	miR160_probe	TGGCATACAGGGAGCCAGGCA

qPCR analyses

Target	Oligo_name	Sequence
AtSN1 Chop-qPCR	AtSN1_Chop-qPCR_F	ACCAACGTGTTGTTGGCCCAGTGGTAA
	AtSN1_Chop-qPCR_R	AAAATAAGTGGTGGTTGTACAAGC
AtSN1 RT-qPCR	AtSN1_RT-qPCR_F	CCAGAAATTCATCTTCTTTGGAAAAG
	AtSN1_RT-qPCR_R	GCCCAGTGGTAAATCTCTCAGATAGA
ONSEN RT-qPCR	ONSEN_qPCR_F	CCACAAGAGGAACCAACGAA
	ONSEN_qPCR_R	TTCGATCATGGAAGACCGG
UBQ10 RT-qPCR	UBQ10_qPCR_F	GGCCTTGTATAATCCCTGATGAATAAG
	UBQ10_qPCR_R	AAAGAGATAACAGGAACGGAAACATAGT

Supplementary Table 2. List of buffers used in this study.

Name	Composition
Extraction Buffer (EB)	0.7 M Sucrose, 0.5 M Tris-HCl (pH 8.0), 5 mM EDTA (pH 8.0), 0.1 M NaCl, 1X Complete ethylenediaminetetraacetic acid (EDTA)-free Protease Inhibitor Cocktail (Roche), 2% β-mercaptoethanol
Resuspension Buffer (RB)	10% glycerol, 3% sodium dodecyl sulphate (SDS), 62.3 mM Tris–HCl (pH 8.0)
Lysis Buffer (LB)	50 mM Tris-HCl (pH 8.0), 50 mM NaCl, 5 mM $MgCl_2$, 1% triton, 1X protease inhibitor cocktail Roche, 1 mM DTT
Wash Buffer (WB)	50 mM Tris-HCl (pH 8.0), 50 mM NaCl, 5 mM $MgCl_2$, 0.1% triton, 1X protease inhibitor cocktail Roche, 1mM DTT
IP buffer (IP)	50 mM Tris-HCl pH 7.5, 150 mM NaCl, 5 mM $MgCl_2$, 10% glycerol, 0.1% NP40, 100 mM phenylmethylsulfonyl fluoride (PMSF) 10 mM Pepstatin, 10mM Bortezomib, 1 mM DTT, 1 Complete Tab protease inhibitor cocktail EDTA-free (Roche)
Running buffer (RB)	25 mM Tris, 192 mM Glycine, 0.1% SDS
Transfer butter (TB)	25 mM Tris, 192 mM Glycine, and 20% ethanol
Western wash buffer (WWB)	20 mM Tris, 150 mM NaCl and 0.1% Tween20 TBS-T

Supplementary Table 3. NRPD1 exclusively conserved regions and functional domains

Exclusively conserved (EC) regions

Name	AtNRPD1 position	Functions	References
EC1	aa 90-105	CLSY docking?	this work
EC2	aa 116-136	CLSY docking	this work
EC3	aa 574-586	unknown	this work
EC4	aa 620-651	Pol IV-RDR2 interaction	this work; (Huang et al., 2021)
EC5	aa 697-716	Pol IV-RDR2 interaction	this work; (Huang et al., 2021)

Selected functional domains and motifs

Name	AtNRPD1 position	Functions	References
Clamp core	aa 1-84	required for Pol IV function	(Ferrafiat et al., 2019)
Clamp core	aa 220-319	required for Pol IV function?	
Clamp head	aa 85-219	CLSY docking	this work
CYC	aa 118-121	CLSY docking	this work; (Ferrafiat et al., 2019)
YPMF	aa 127-132	CLSY docking	this work
Active site	aa 447,449,451	Metal-A binding site	(Onodera et al., 2005; Haag et al., 2009; Lahmy et al., 2009)
Funnel helices	aa 621-732	Pol IV-RDR2 interaction	(Huang et al., 2021)
Funnel helix tip	aa 658-693	Not required for Pol IV function	(Huang et al., 2021)
