# Supplementary material for: CLSY docking to Pol IV requires a conserved domain critical for small RNA biogenesis and transposon silencing
Source: Nat Commun. 2024 Nov 27;15:10298. doi: 10.1038/s41467-024-54268-0 (PMC11603163; doi:10.1038/s41467-024-54268-0)
Supplement: Supplementary file 2 — Description of Additional Supplementary Files [file 41467_2024_54268_MOESM2_ESM.pdf]

Description of Additional Supplementary files

Supplementary Data 1. List of the plant RNA polymerase subunits used for multiple sequence alignment and phylogenetic analyses (Fig. 1c and 1d, and Supplementary Figs. 1 and 2).

Supplementary Data 2. Spectral counts of proteins identified by IP-MS of Pol IV complexes containing NRPD1-3xF_{WT}, or the NRPD1-3x$F_{AAA\text{-}YPMF}$ or NRPD1-3x$F_{CYC\text{-}AAAA}$ variants. The adj. p-values derive from a quasi-likelihood negative binomial generalized log-linear model in IPinquiry4.

Supplementary Data 3. Spectral counts of proteins enriched in CLSY1, CLSY3 and CLSY4 3xF IP-MS experiments. The adj. p-values derive from a quasi-likelihood negative binomial generalized log-linear model in IPinquiry4.

Supplementary Data 4. Spectral counts of proteins copurifying with 3xHA-CLSY1 in NRPD1-3xF_{WT} or NRPD1-3x$F_{AAA\text{-}YPMF}$ motif variant contexts identified by IP-MS. The adj. p-values derive from a quasi-likelihood negative binomial generalized log-linear model in IPinquiry4.

Supplementary Data 5. Summary of smRNA-seq data processing.
