# Supplementary material for: CLSY docking to Pol IV requires a conserved domain critical for small RNA biogenesis and transposon silencing
Source: Nat Commun. 2024 Nov 27;15:10298. doi: 10.1038/s41467-024-54268-0 (PMC11603163; doi:10.1038/s41467-024-54268-0)
Supplement: Supplementary file 8 — Reporting Summary [file 41467_2024_54268_MOESM8_ESM.pdf]

nature portfolio

Corresponding author(s): Todd Blevins and Julie A. Law

Last updated by author(s): October 10, 2024

Reporting Summary

Nature Portfolio wishes to improve the reproducibility of the work that we publish. This form provides structure for consistency and transparency in reporting. For further information on Nature Portfolio policies, see our Editorial Policies and the Editorial Policy Checklist.

Statistics

For all statistical analyses, confirm that the following items are present in the figure legend, table legend, main text, or Methods section.

n/a	Confirmed	
☐	☒	The exact sample size (*n*) for each experimental group/condition, given as a discrete number and unit of measurement
☐	☒	A statement on whether measurements were taken from distinct samples or whether the same sample was measured repeatedly
☐	☒	The statistical test(s) used AND whether they are one- or two-sided<br>*Only common tests should be described solely by name; describe more complex techniques in the Methods section.*
☒	☐	A description of all covariates tested
☒	☐	A description of any assumptions or corrections, such as tests of normality and adjustment for multiple comparisons
☐	☒	A full description of the statistical parameters including central tendency (e.g. means) or other basic estimates (e.g. regression coefficient) AND variation (e.g. standard deviation) or associated estimates of uncertainty (e.g. confidence intervals)
☐	☒	For null hypothesis testing, the test statistic (e.g. *F*, *t*, *r*) with confidence intervals, effect sizes, degrees of freedom and *P* value noted<br>*Give P values as exact values whenever suitable.*
☒	☐	For Bayesian analysis, information on the choice of priors and Markov chain Monte Carlo settings
☒	☐	For hierarchical and complex designs, identification of the appropriate level for tests and full reporting of outcomes
☒	☐	Estimates of effect sizes (e.g. Cohen's *d*, Pearson's *r*), indicating how they were calculated

Our web collection on statistics for biologists contains articles on many of the points above.

Software and code

Policy information about availability of computer code

Data collection	n/a
Data analysis	Code availability<br>=============<br>Multiple standard software tools were used: Mascot (v2.6.2), Proline (v2.0), R (v4.2.3), R package IPinquiry4 (Kuhn et al. 2023), cutadapt (v1.18), ShortStack (v3.8.5), bamtools (v2.5.1), HOMER (v4.10), ggplot2 (v3.5.0), reshape2 (v1.4.4), and DESeq2 (v1.42.1) in addition to the custom scripts JSON_findPerfectMatches_and_TerminalMisMatches_v3 (Zhou et al. 2018) and splitTagDirectoryByLength.dev2.pl (Zhou et al. 2018). The latter custom bioinformatics code used for processing, analyzing and visualizing the results is available at https://github.com/toddblev/Felgines_Rymen_Martins_2024 and the version used was also deposited at the Zenodo database under the DOI https://doi.org/10.5281/zenodo.13890517 [https://zenodo.org/doi/10.5281/zenodo.13890517].

For manuscripts utilizing custom algorithms or software that are central to the research but not yet described in published literature, software must be made available to editors and reviewers. We strongly encourage code deposition in a community repository (e.g. GitHub). See the Nature Portfolio guidelines for submitting code & software for further information.

Data

Policy information about availability of data

All manuscripts must include a data availability statement. This statement should provide the following information, where applicable:

- Accession codes, unique identifiers, or web links for publicly available datasets
- A description of any restrictions on data availability
- For clinical datasets or third party data, please ensure that the statement adheres to our policy

Data availability
=============
The source data for Figs. 2, 4, and Supplementary Figs. 5, 7, are provided as a Source Data file. The IP-MS datasets obtained in this work are available at the PRIDE server via the identifier PXD047743 [https://www.ebi.ac.uk/pride/archive/projects/PXD047743], and at the MassIVE server via identifier MSV000093500 [ftp://massive.ucsd.edu/v06/MSV000093500/]. The Illumina smRNA-seq datasets collected for the study were deposited in the NCBI Gene Expression Omnibus (GEO) and are available via the accession numbers GSE242191 [https://www.ncbi.nlm.nih.gov/geo/query/acc.cgi?acc=GSE242191] and GSE278181 [https://www.ncbi.nlm.nih.gov/geo/query/acc.cgi?acc=GSE278181]. Previously published smRNA-seq datasets, reanalyzed here, are described in Supplementary Data 5. All other data supporting this study are available from the corresponding authors upon reasonable request.

Research involving human participants, their data, or biological material

Policy information about studies with human participants or human data. See also policy information about sex, gender (identity/presentation), and sexual orientation and race, ethnicity and racism.

Reporting on sex and gender	n/a
Reporting on race, ethnicity, or other socially relevant groupings	n/a
Population characteristics	n/a
Recruitment	n/a
Ethics oversight	n/a

Note that full information on the approval of the study protocol must also be provided in the manuscript.

Field-specific reporting

Please select the one below that is the best fit for your research. If you are not sure, read the appropriate sections before making your selection.

☒ Life sciences ☐ Behavioural & social sciences ☐ Ecological, evolutionary & environmental sciences

For a reference copy of the document with all sections, see nature.com/documents/nr-reporting-summary-flat.pdf

Life sciences study design

All studies must disclose on these points even when the disclosure is negative.

Sample size	No experiments were conducted that required consideration of a sample size.
Data exclusions	Data was not excluded from data visualizations, other than the omission of smaller protein subunits in Pol IV-RDR2 structural models to better display the largest subunit of Pol IV (NRPD1), its interface with RDR2, and the arrangements of NRPD1's evolutionarily conserved domains, as described in the legend of Fig. 1.
Replication	- Immunoprecipitation-mass spectrometry (IP-MS): For the NRPD1-3xF IP-MS experiments (Fig 2B,C,D), 2 technical replicates were performed on 3 independent lines per variant (WT, AAA-YPMF, CYC-AAAA) for a total of 6 datasets per NRPD1-3xF variant. For the 3xF CLSY IP-MS experiments (Fig 1A,B), 3 independent IPMS experiments (b#) were performed on 2 to 3 independent lines per CLSY (L#) with 1 to 2 technical replicates. For the 3xHA-CLSY1 IP-MS experiments, triplicate batches of the two different genotypes in Fig 1D were analyzed.<br>- smRNA-seq (Fig. 4A,B,S7): For each genotype, two biological replicates were compared to other such biological duplicates across a large panel of mutant genotypes and to matched wild-type controls.<br>- Western blot detection of CLSY3-3xF-BLRP co-IP with NRPD1-3xF (Fig. 2E) was replicated as shown in Fig. S5C. Western blot detections of NRPD1-3xF expression in each of the 3 independent lines per variant are provided (Fig. S5A), along with western blot analyses of each of the two technical replicate IPs for these lines (Fig. S5B).<br>- qPCR assays with technical replicates: In the RT-qPCR assays, n = 6 for AtSN1 (Fig. 4D) and n = 3 for ONSEN (Fig. 4E). In the Chop-qPCR analysis, n = 2 for the Col-0, nrpd1 and clsy quad samples, and n = 4 for the NRPD1-3xF lines (Fig. 4C).
Randomization	No assays were conducted that required randomization.

Blinding	Blinding was not relevant to this study as nearly all analyses were based on standard molecular assays, immunoprecipitation-mass spectrometry experiments and Illumina sequencing, and thus were not prone to individual/subjective biases.

Reporting for specific materials, systems and methods

We require information from authors about some types of materials, experimental systems and methods used in many studies. Here, indicate whether each material, system or method listed is relevant to your study. If you are not sure if a list item applies to your research, read the appropriate section before selecting a response.

Materials & experimental systems

n/a	Involved in the study	
☐	☒	Antibodies
☒	☐	Eukaryotic cell lines
☒	☐	Palaeontology and archaeology
☒	☐	Animals and other organisms
☒	☐	Clinical data
☒	☐	Dual use research of concern
☐	☒	Plants

Methods

n/a	Involved in the study	
☒	☐	ChIP-seq
☒	☐	Flow cytometry
☒	☐	MRI-based neuroimaging

Antibodies

Antibodies used	anti-FLAG beads (Miltenyi Biotec #130-101-591)<br>anti-HA beads (Miltenyi Biotec #130-091-122)<br>anti-FLAG beads (Sigma #M8823)<br>Streptavidin beads (M-280 Dynabeads #11205D)<br>anti-NRPD1 (see Ferrafiat et al. 2019)<br>anti-NRPD2 (see Ferrafiat et al. 2019)<br>anti-FLAG-HRP antibody (Sigma #A8592)<br>anti-HA-HRP antibody (Sigma #H6533)
Validation	Anti-NRPD1 and anti-NRPD2 antibodies were each validated by western blot analyses of total protein extracted from wild-type, nrpd1-3, or nrpd2-2 null mutant Arabidopsis thaliana plants, respectively, showing that an expected size protein was detected only in the wild-type but not in the corresponding mutant sample (see Ferrafiat et al. 2019).

Plants

Seed stocks	Arabidopsis thaliana: nrpd1-3 (SALK_128428), nrpd1-4 (SALK_083051), clsy1-7 (SALK_018319), clsy2-1 (GABI-Kat line 554E02), clsy3-1 (SALK_040366), clsy4-1 (SALK_003876); nrpd1-49, nrpd1-50 and nrpd1-51 point mutations were described by Ferrafiat et al. 2019.
Novel plant genotypes	Arabidopsis thaliana: NRPD1p::NRPD1-3xF lines expressing either wild-type (NRPD1-3xF WT) or mutant (NRPD1-3xF AAA-YPMF or NRPD1-3xF CYC-AAAA) forms of the NRPD1-3xF protein in the nrpd1-3 mutant background.<br>The following lines expressing CLSY1, CLSY3 or CLSY4 were used in this study: pC1::3xF-CLSY1 in clsy1-7 (LR39-2-5), pC1::3xF-CLSY1-wt in clsy1-7 (LR136-119-6), pC3::CLSY3-3xF in clsy3-1 (LR31-22-4), pC3::CLSY3-3xF-mut in clsy3-1 (LR154-196-7), pC4::3xF-CLSY4 in clsy4-1 (LR59-6-2), pC4::CLSY4-3xF in clsy4-1 (LR35-2-3), and pC4::CLSY4-3xF in clsy4-1 (LR35-4-3).<br>Lines with CLSY1p::3xHA-CLSY1 transformed into the NRPD1-3xF WT or NRPD1-3xF AAA-YPMF background.
Authentication	*Describe any authentication procedures for each seed stock used or novel genotype generated. Describe any experiments used to assess the effect of a mutation and, where applicable, how potential secondary effects (e.g. second site T-DNA insertions, mosiacism, off-target gene editing) were examined.*
